# 4-Acetyl­pyridinium hydrogen sulfate

**DOI:** 10.1107/S1600536809035417

**Published:** 2009-09-09

**Authors:** Xue-qun Fu

**Affiliations:** a Ordered Matter Science Research Center, Southeast University, Nanjing 210096, People’s Republic of China

## Abstract

The crystal structure of the title compound, C_7_H_8_NO^+^·HSO_4_^−^, consists of O—H⋯Ohydrogen-bonded extended chains of hydrogen sulfate anions. Each hydrogen sulfate anion is furthermore connected to one 4-acetyl­pyridinium cation *via* a hydrogen bond of the N—H⋯O type.

## Related literature

For the synthesis of 4-acetyl­pyridine, see: Piner *et al.* (1934 ▶). For the crystal structure of an adduct of 4-acetyl­pyridine with penta­chloro­phenol, see: Majerz *et al.* (1991 ▶). For the crystal structures of Zn and Ni complexes of 4-acetyl­pyridine, see: Pang *et al.* (1994 ▶); Steffen *et al.* (1977 ▶).
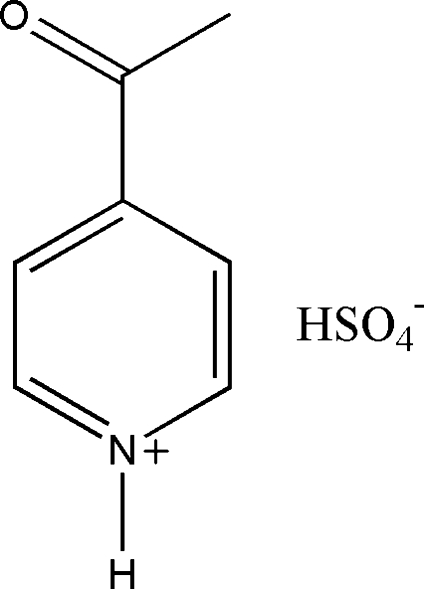


## Experimental

### 

#### Crystal data

C_7_H_8_NO^+^·HSO_4_^−^
*M* *_r_* = 219.21
Orthorhombic, 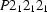

*a* = 4.6454 (9) Å
*b* = 9.597 (2) Å
*c* = 21.310 (4) Å
*V* = 950.1 (3) Å^3^
*Z* = 4
Mo *K*α radiation
μ = 0.34 mm^−1^
*T* = 298 K
0.20 × 0.20 × 0.20 mm

#### Data collection

Rigaku SCXmini diffractometer
Absorption correction: multi-scan (*CrystalClear*; Rigaku, 2005 ▶) *T*_min_ = 0.935, *T*_max_ = 0.935
9843 measured reflections
2156 independent reflections
1622 reflections with *I* > 2σ(*I*)
*R*_int_ = 0.077

#### Refinement

*R*[*F* ^2^ > 2σ(*F* ^2^)] = 0.053
*wR*(*F* ^2^) = 0.170
*S* = 0.93
2156 reflections
129 parameters
H-atom parameters constrained
Δρ_max_ = 0.38 e Å^−3^
Δρ_min_ = −0.19 e Å^−3^
Absolute structure: Flack (1983 ▶), 860 Friedel pairs
Flack parameter: 0.2 (2)

### 

Data collection: *CrystalClear* (Rigaku, 2005 ▶); cell refinement: *CrystalClear*; data reduction: *CrystalClear*; program(s) used to solve structure: *SHELXS97* (Sheldrick, 2008 ▶); program(s) used to refine structure: *SHELXL97* (Sheldrick, 2008 ▶); molecular graphics: *SHELXTL* (Sheldrick, 2008 ▶); software used to prepare material for publication: *PRPKAPPA* (Ferguson, 1999 ▶).

## Supplementary Material

Crystal structure: contains datablocks I, global. DOI: 10.1107/S1600536809035417/im2126sup1.cif

Structure factors: contains datablocks I. DOI: 10.1107/S1600536809035417/im2126Isup2.hkl

Additional supplementary materials:  crystallographic information; 3D view; checkCIF report

## Figures and Tables

**Table 1:** Hydrogen-bond geometry (Å, °)

*D*—H⋯*A*	*D*—H	H⋯*A*	*D*⋯*A*	*D*—H⋯*A*
N1—H1*B*⋯O1^i^	0.86	1.92	2.772 (5)	172
O3—H3⋯O2^ii^	0.82	1.76	2.565 (5)	166

Symmetry codes: (i) 
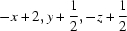
; (ii) 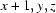.

**Fig. 1.**
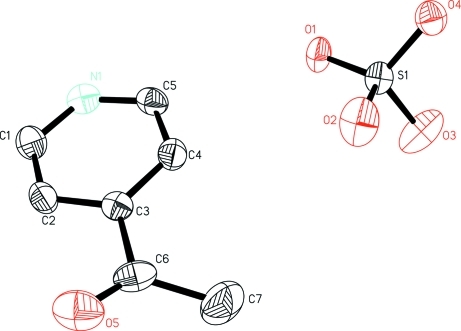
Molecular structure of the title compound with the atomic numbering scheme. Displacement ellipsoids are drawn at the 30% probability level and all H atoms have been omitted for clarity.

**Fig. 2.**
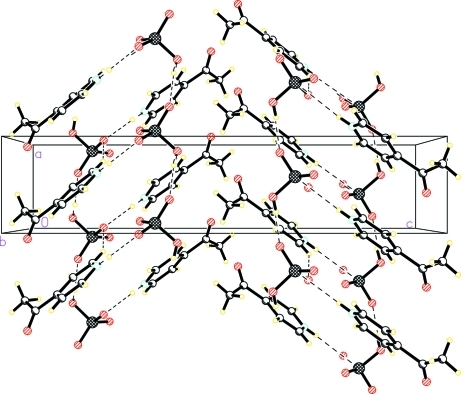
Crystal packing of the title compound, stacking along the b axis. Dashed lines indicate hydrogen bonds.

## supplementary crystallographic information

### Comment

4-Acetylpyridine may be used as a ligand in coordination compounds *e.g.* with Zn (Steffen & Palenik, 1977) or Ni (Pang *et al.*, 1994). The crystal structure of 4-acetylpyridine together with pentachlorophenol is also known (Majerz *et al.*, 1991).

The asymmetric unit of the title compound contains one 4-acetylpyridinium cation and one hydrogen sulfate anion (Fig 1). In the anion, the bond length of S1—O3 is 1.553 (6) Å compared to the average bond length of 1.438 (5) Å of the other S1—O bonds. It is therefore reasonable that the hydrogen atom of hydrogen sulfate is bonded to O3. The supramolecular structure consists of infinite chains of anions with one cation linked to each anion *via* an additional hydrogen bond (N1—H1B···O1 2.774 (8) Å, Fig 2).

### Experimental

4-Acetylpyridine was obtained according to the method described by Piner (1934). Reaction of equimolar amounts of 4-acetylpyridine and H_2_SO_4 _produced a precipitate. This was filtered off, dried and dissolved in 96% ethanol from which single crystals were grown by slow evaporation of the solvent at room temperature.

### Refinement

The H atom connected to O3 was discernible from difference electron-density map. Nevertheless, it was placed to the ideal position, with S1—O3—H angle tetrahedral, allowing the H atom to ride on the immediately preceding atom O3 and rotate about the S1—O3 bond, refined in a riding atom approximation with a constrained bond length of O—H = 0.82 Å. Positional parameters of the other H atoms were calculated geometrically with C_ar_–H = 0.93 Å and C_Me_–H = 0.96 Å and were allowed to ride on the corresponding C atoms with *U*_iso_(H) = 1.2 *U*_eq_(C). In the absence of significant anomalous dispersion effects, 860 Friedel pairs were merged.

### Crystal data

**Table:**

C_7_H_8_NO^+^·HSO_4_^−^	*F*(000) = 456
*M**_r_* = 219.21	*D*_x_ = 1.533 Mg m^−^^3^
Orthorhombic, *P*2_1_2_1_2_1_	Mo *K*α radiation, λ = 0.71073 Å
Hall symbol: P 2ac 2ab	Cell parameters from 4231 reflections
*a* = 4.6454 (9) Å	θ = 3.6–27.6°
*b* = 9.597 (2) Å	µ = 0.34 mm^−^^1^
*c* = 21.310 (4) Å	*T* = 298 K
*V* = 950.1 (3) Å^3^	Prism, colourless
*Z* = 4	0.20 × 0.20 × 0.20 mm

### Data collection

**Table:**

Rigaku SCXmini diffractometer	2156 independent reflections
Radiation source: fine-focus sealed tube	1622 reflections with *I* > 2σ(*I*)
graphite	*R*_int_ = 0.077
Detector resolution: 13.6612 pixels mm^-1^	θ_max_ = 27.5°, θ_min_ = 3.6°
ω scans	*h* = −6→6
Absorption correction: multi-scan (*CrystalClear*; Rigaku, 2005)	*k* = −12→12
*T*_min_ = 0.935, *T*_max_ = 0.935	*l* = −27→27
9843 measured reflections	

### Refinement

**Table:**

Refinement on *F*^2^	Secondary atom site location: difference Fourier map
Least-squares matrix: full	Hydrogen site location: inferred from neighbouring sites
*R*[*F*^2^ > 2σ(*F*^2^)] = 0.053	H-atom parameters constrained
*wR*(*F*^2^) = 0.170	*w* = 1/[σ^2^(*F*_o_^2^) + (0.1249*P*)^2^ + 1.8027*P*] where *P* = (*F*_o_^2^ + 2*F*_c_^2^)/3
*S* = 0.93	(Δ/σ)_max_ < 0.001
2156 reflections	Δρ_max_ = 0.38 e Å^−^^3^
129 parameters	Δρ_min_ = −0.19 e Å^−^^3^
0 restraints	Absolute structure: Flack (1983), 860 Friedel pairs
Primary atom site location: structure-invariant direct methods	Flack parameter: 0.2 (2)

### Special details

**Table:**

Geometry. All e.s.d.'s (except the e.s.d. in the dihedral angle between two l.s. planes) are estimated using the full covariance matrix. The cell e.s.d.'s are taken into account individually in the estimation of e.s.d.'s in distances, angles and torsion angles; correlations between e.s.d.'s in cell parameters are only used when they are defined by crystal symmetry. An approximate (isotropic) treatment of cell e.s.d.'s is used for estimating e.s.d.'s involving l.s. planes.
Refinement. Refinement of *F*^2^ against ALL reflections. The weighted *R*-factor *wR* and goodness of fit *S* are based on *F*^2^, conventional *R*-factors *R* are based on *F*, with *F* set to zero for negative *F*^2^. The threshold expression of *F*^2^ > σ(*F*^2^) is used only for calculating *R*-factors(gt) *etc*. and is not relevant to the choice of reflections for refinement. *R*-factors based on *F*^2^ are statistically about twice as large as those based on *F*, and *R*- factors based on ALL data will be even larger.

### Fractional atomic coordinates and isotropic or equivalent isotropic displacement parameters (Å^2^)

**Table:**

	*x*	*y*	*z*	*U*_iso_*/*U*_eq_	
S1	0.9101 (2)	0.18577 (10)	0.16383 (5)	0.0475 (3)	
O5	−0.0805 (8)	0.6665 (5)	0.03394 (17)	0.0853 (12)	
O1	1.0236 (7)	0.3041 (3)	0.19787 (16)	0.0655 (9)	
N1	0.6303 (8)	0.7352 (4)	0.20080 (17)	0.0573 (9)	
H1B	0.7336	0.7648	0.2315	0.069*	
C3	0.3084 (8)	0.6431 (4)	0.10302 (18)	0.0457 (9)	
C1	0.4610 (10)	0.8250 (5)	0.1713 (2)	0.0614 (11)	
H1A	0.4549	0.9175	0.1842	0.074*	
C6	0.1250 (13)	0.5981 (6)	0.0476 (2)	0.0634 (13)	
C5	0.6477 (10)	0.6005 (5)	0.1849 (2)	0.0548 (11)	
H5A	0.7674	0.5403	0.2070	0.066*	
C4	0.4852 (9)	0.5528 (5)	0.1353 (2)	0.0544 (11)	
H4A	0.4949	0.4596	0.1236	0.065*	
C2	0.2960 (10)	0.7819 (4)	0.1223 (2)	0.0546 (10)	
H2A	0.1759	0.8446	0.1017	0.066*	
O2	0.6532 (7)	0.2155 (4)	0.1291 (2)	0.0858 (12)	
O3	1.1265 (8)	0.1454 (6)	0.1117 (2)	0.0968 (15)	
H3	1.2838	0.1802	0.1194	0.145*	
O4	0.8942 (13)	0.0646 (4)	0.2026 (2)	0.1068 (15)	
C7	0.2139 (18)	0.4694 (7)	0.0132 (3)	0.106 (2)	
H7A	0.0795	0.4510	−0.0200	0.160*	
H7B	0.2166	0.3919	0.0417	0.160*	
H7C	0.4026	0.4824	−0.0042	0.160*	

### Atomic displacement parameters (Å^2^)

**Table:**

	*U*^11^	*U*^22^	*U*^33^	*U*^12^	*U*^13^	*U*^23^
S1	0.0356 (4)	0.0481 (5)	0.0588 (5)	−0.0017 (4)	−0.0011 (4)	−0.0066 (5)
O5	0.058 (2)	0.121 (3)	0.077 (2)	−0.004 (3)	−0.0169 (19)	0.008 (2)
O1	0.069 (2)	0.0471 (16)	0.080 (2)	0.0051 (15)	−0.0109 (17)	−0.0183 (15)
N1	0.046 (2)	0.073 (2)	0.0531 (19)	−0.0074 (18)	0.0000 (17)	−0.0004 (17)
C3	0.0372 (18)	0.054 (2)	0.0459 (19)	−0.0021 (16)	0.0076 (16)	0.0062 (17)
C1	0.063 (3)	0.049 (2)	0.072 (3)	−0.003 (2)	0.002 (2)	0.000 (2)
C6	0.059 (3)	0.083 (3)	0.048 (2)	−0.016 (3)	0.009 (2)	0.003 (2)
C5	0.044 (2)	0.066 (3)	0.054 (2)	0.007 (2)	−0.0010 (19)	0.0119 (19)
C4	0.060 (3)	0.045 (2)	0.058 (2)	0.0030 (19)	0.014 (2)	0.0029 (18)
C2	0.055 (2)	0.047 (2)	0.062 (2)	0.0078 (19)	0.001 (2)	0.0075 (19)
O2	0.0428 (18)	0.096 (3)	0.118 (3)	0.0099 (17)	−0.021 (2)	−0.021 (2)
O3	0.0471 (19)	0.145 (4)	0.098 (3)	−0.010 (2)	0.001 (2)	−0.060 (3)
O4	0.137 (4)	0.073 (2)	0.110 (3)	−0.039 (3)	−0.028 (3)	0.022 (2)
C7	0.128 (6)	0.110 (5)	0.081 (4)	0.002 (5)	−0.020 (4)	−0.039 (4)

### Geometric parameters (Å, °)

**Table:**

S1—O4	1.429 (4)	C1—C2	1.360 (6)
S1—O2	1.433 (4)	C1—H1A	0.9300
S1—O1	1.447 (3)	C6—C7	1.495 (8)
S1—O3	1.547 (4)	C5—C4	1.378 (6)
O5—C6	1.194 (7)	C5—H5A	0.9300
N1—C1	1.325 (6)	C4—H4A	0.9300
N1—C5	1.339 (6)	C2—H2A	0.9300
N1—H1B	0.8600	O3—H3	0.8200
C3—C4	1.378 (6)	C7—H7A	0.9600
C3—C2	1.395 (6)	C7—H7B	0.9600
C3—C6	1.519 (6)	C7—H7C	0.9600
			
O4—S1—O2	114.7 (3)	C7—C6—C3	117.5 (5)
O4—S1—O1	111.6 (2)	N1—C5—C4	118.8 (4)
O2—S1—O1	113.9 (2)	N1—C5—H5A	120.6
O4—S1—O3	104.2 (3)	C4—C5—H5A	120.6
O2—S1—O3	102.7 (2)	C5—C4—C3	120.0 (4)
O1—S1—O3	108.7 (2)	C5—C4—H4A	120.0
C1—N1—C5	122.9 (4)	C3—C4—H4A	120.0
C1—N1—H1B	118.5	C1—C2—C3	119.5 (4)
C5—N1—H1B	118.5	C1—C2—H2A	120.2
C4—C3—C2	118.6 (4)	C3—C2—H2A	120.2
C4—C3—C6	123.0 (4)	S1—O3—H3	109.5
C2—C3—C6	118.5 (4)	C6—C7—H7A	109.5
N1—C1—C2	120.1 (4)	C6—C7—H7B	109.5
N1—C1—H1A	120.0	H7A—C7—H7B	109.5
C2—C1—H1A	120.0	C6—C7—H7C	109.5
O5—C6—C7	123.8 (5)	H7A—C7—H7C	109.5
O5—C6—C3	118.8 (5)	H7B—C7—H7C	109.5
			
C5—N1—C1—C2	−0.4 (7)	N1—C5—C4—C3	−0.1 (6)
C4—C3—C6—O5	157.9 (4)	C2—C3—C4—C5	−0.8 (6)
C2—C3—C6—O5	−21.8 (6)	C6—C3—C4—C5	179.5 (4)
C4—C3—C6—C7	−21.9 (6)	N1—C1—C2—C3	−0.5 (7)
C2—C3—C6—C7	158.4 (5)	C4—C3—C2—C1	1.1 (6)
C1—N1—C5—C4	0.7 (7)	C6—C3—C2—C1	−179.2 (4)

### Hydrogen-bond geometry (Å, °)

**Table:**

*D*—H···*A*	*D*—H	H···*A*	*D*···*A*	*D*—H···*A*
N1—H1B···O1^i^	0.86	1.92	2.772 (5)	172
O3—H3···O2^ii^	0.82	1.76	2.565 (5)	166

Symmetry codes: (i) −*x*+2, *y*+1/2, −*z*+1/2; (ii) *x*+1, *y*, *z*.
